# Bioinformatics Analysis of Genes and Mechanisms in Postherpetic Neuralgia

**DOI:** 10.1155/2020/1380504

**Published:** 2020-09-24

**Authors:** Yong Qiu, Meng-Lei Hao, Xu-Tao Cheng, Zhen Hua

**Affiliations:** ^1^Anesthesiology Department, Beijing Hospital, National Center of Gerontology, Institute of Geriatric Medicine, Chinese Academy of Medical Sciences, No. 1 Dahua Road, Dong Dan, Beijing 100730, China; ^2^Department of Geriatric Medicine, Affiliated Hospital of Qinghai University, No. 29 Tongren Road, Xining 810001, Qinghai Province, China

## Abstract

**Objective:**

Elderly patients are prone to postherpetic neuralgia (PHN), which may cause anxiety, depression, and sleep disorders and reduce quality of life. As a result, the life quality of patients was seriously reduced. However, the pathogenesis of PHN has not been fully elucidated, and current treatments remain inadequate. Therefore, it is important to explore the molecular mechanism of PHN.

**Methods:**

We analyzed the GSE64345 dataset, which includes gene expression from the ipsilateral dorsal root ganglia (DRG) of PHN model rats. Differentially expressed genes (DEGs) were identified and analyzed by Gene Ontology. Protein-protein interaction (PPI) network was constructed. The miRNA associated with neuropathic pain and inflammation was found in miRNet. Hub genes were identified and analyzed in Comparative Toxicogenomics Database (CTD). miRNA-mRNA networks associated with PHN were constructed.

**Results:**

A total of 116 genes were up-regulated in the DRG of PHN rats, and 135 genes were down-regulated. Functional analysis revealed that variations were predominantly enriched for genes involved in neuroactive ligand-receptor interactions, the Jak-STAT signaling pathway, and calcium channel activity. Eleven and thirty-one miRNAs associated with neuropathic pain and inflammation, respectively, were found. Eight hub genes (S1PR1, OPRM1, PDYN, CXCL3, S1PR5, TBX5, TNNI3, MYL7, PTGDR2, and FBXW2) associated with PHN were identified.

**Conclusions:**

Bioinformatics analysis is a useful tool to explore the mechanism and pathogenesis of PHN. The identified hub genes may participate in the onset and development of PHN and serve as therapeutic targets.

## 1. Introduction

Postherpetic neuralgia (PHN) is an intractable condition, characterized by persistent or intermittent burning, tingling, or sharp pain, that lasts more than 4 months [1] and affects patients' quality of life [2]. It is estimated that the incidence of PHN was 3.9–42.0/100,000 person-years [3]. A study involving 1358 patients with acute herpes zoster found that the incidence of herpes zoster-related pain at 6 months was 9% [4]. Furthermore, PHN may cause complications such as anxiety, depression, and sleep disorders, which may reduce the life expectancy of elderly patients [5]. Therefore, early diagnosis and timely treatment are very important for elderly patients [1]. However, the pathogenesis of PHN has not been fully elucidated. Inflammation, neuroinflammation, loss of axons and myelin sheath of sensory nerve roots, fibrosis, and central sensitization may be involved [6, 7], and the dorsal root ganglion (DRG) may play an important role in the occurrence, development, and treatment of PHN [8, 9]. Gabapentin, pregabalin, and opioids can improve the symptoms of PHN, but their therapeutic effects are still far from satisfactory [10]. Mirogabalin and the angiotensin II; type 2 receptor antagonist EMA401 may be effective in relieving the symptoms of PHN, but additional clinical investigation is warranted [11, 12]. Therefore, we aimed to further explore the pathogenesis of PHN and identify specific molecular targets.

Gene sequencing and bioinformatics analysis are widely used in the study of the molecular mechanism of diseases. Tang found several DEGs upon analysis of the transcriptional data of DRG in spared nerve injury (SNI) model mice and determined that mir-16-5p may be involved in the pathogenesis of neuropathic pain (NP) [13]. Following an analysis of the transcriptional data of patients with fibromyalgia, Qiu found evidence that CD38 and GATM are involved in the occurrence and development of this disease through regulation of ion channels and inflammatory pathways, which provides new insights into the molecular mechanisms [14]. Furthermore, Guedon successfully constructed a rat model of PHN using Varicella Zoster Virus (VZV) and found several DEGs in DRG sequencing data, including the abnormal expression of CGRP and TRPV1 [15]. Another lab found several abnormally expressed miRNAs and circRNAs in the skin lesions of patients with PHN [16]. However, these data still need additional clinical interpretation.

Through bioinformatics analysis, we found DEGs between the DRG of PHN rats and normal control rats. Hub genes were identified and analyzed in the Comparative Toxicogenomics Database (CTD), and their roles in PHN were preliminarily analyzed.

## 2. Materials and Methods

### 2.1. PHN Dataset Selection

One expression profiling dataset [GSE64345 (GPL1355 platform)] was downloaded from the GEO database (http://www.ncbi.nlm.nih.gov/geo), an open-source platform for the retrieving gene expression data [17]. The GSE64345 dataset includes gene expression in the ipsilateral DRG of three PHN model rats (male, Sprague Dawley rats) and three normal controls. Varicella zoster virus- (VZV-) infected MEWO (human melanoma cell line MeWo) human cells were used to transmit VZV to rodents. Controls (uninfected MEWO cells) or VZV-infected MEWO cells were inoculated into the glabrous region of the right rear footpad of male Sprague Dawley rats. After the development of ipsilateral nocifensive behaviour in the VZV-infected animals, the ipsilateral DRG (L4, 5) from infected and control animals were taken for microarray analysis [15].

### 2.2. DEGs Identification

GEO2R (https://www.ncbi.nlm.nih.gov/geo/geo2r/), an online tool based on GEOquery and limma *R* packages, was used to identify DEGs between DRGs of PHN rats and normal control [18]. GEO2R may also be used to distinguish DEGs between DRGs of PHN rats and normal control. Results for which *P* value <0.05 and Fold change (FC) > 1 or FC＜−1 were considered statistically significant. Volcano diagrams were delineated by SangerBox software based on *R* language (http://sangerbox.com/).

### 2.3. GO and KEGG Analysis of DEGs

The Database for Annotation, Visualization, and Integrated Discovery (DAVID) (https://david.ncifcrf.gov/home.jsp; version 6.8) is a useful annotation function tool [19]. Gene Ontology (GO) covers biological process (BP), cellular component (CC), and molecular function (MF). In addition, Encyclopedia of Genes and Genomes (KEGG) is widely used. GO and KEGG analysis of DEGs were performed in DAVID. Background: Rattus norvegicus. Thresholds: count 2, EASE 0.1. Results for which *P* < 0.05 were considered statistically significant. Top 20 of BP, CC, MF, and top 7 KEGG enrichments were selected and visualized. The process and pathway enrichment analysis were performed by Metascape (http://metascape.org/gp/index.html), a powerful annotation analysis tool for gene function [20]. Thresholds: *P*-value cutoff 0.01, min enrichment 1.5.

### 2.4. PPI Network Construction

The Search Tool for the Retrieval of Interacting Genes (STRING) (http://string.embl.de/) was used to construct a PPI network for the DEGs, which was visualized in Cytoscape (version 3.6.1) [21]. The Molecular Complex Detection tool (MCODE) was then used to screen and identify the most significant module in the network. The criteria were MCODE scores >2, node score cutoff = 0.2, and degree of cutoff = 2.

### 2.5. Neuropathic Pain and Inflammation-Related miRNAs and Target Genes

The miRNAs associated with inflammation and neuropathic pain were identified in miRNet, a web-based tool that can predict the miRNA of many diseases based on high-quality data (http://www.mirnet.ca) [22]. Then, the miRNA related genes were performed. Common genes between the predicted genes and DEGs of dataset GSE64345 were identified.

### 2.6. PHN Associated Genes

Using the Comparative Toxicogenomics Database (CTD), which can effectively predict the correlation between diseases, drugs, and genes (http://ctdbase.org/) [23], genes associated with PHN were identified. Common genes were found between PHN associated genes and DEGs of dataset GSE64345.

### 2.7. Hub Gene Identification

The common genes between MCODE genes and the NP-related miRNA predicted genes, inflammation-related miRNA predicted genes, and PHN-associated genes were identified. These common genes were hub genes.

### 2.8. Hub Gene Analysis in CTD

Firstly, the interaction of hub gene and signs and symptoms, mental disorder, and nervous system diseases were analyzed. Then, the intersecting genes among DEGs of GSE64345, PHN-related genes, and genes that inferred with common drugs (Pregabalin, gabapentin, amitriptyline, duloxetine, venlafaxine, and tramadol) used to treat PHN were identified. Since the pathogenesis of PHN has not been elucidated, mechanisms such as central sensitization may be involved [7]. The localization of hub gene in brain cells was explored in Brain RNA-seq, an RNA-sequencing transcriptome, and splicing database of glia and neurons [24].

### 2.9. miRNA-mRNA Network Construction and lncRNA Prediction

The miRNA-mRNA networks of inflammation-related miRNA, NP-related miRNA, and PHN-associated genes were constructed. NP-related miRNA hsa-mir-150-5p and hsa-mir-134-5p predicted lncRNAs were identified and the lncRNA-miRNA network were constructed. Additionally, the miRNA-mRNA networks of inflammation-related miRNA, NP-related miRNA, and PHN-associated genes were constructed.

## 3. Results

### 3.1. DEGs between PHN and Normal Control DRGs

**Figure 1:**
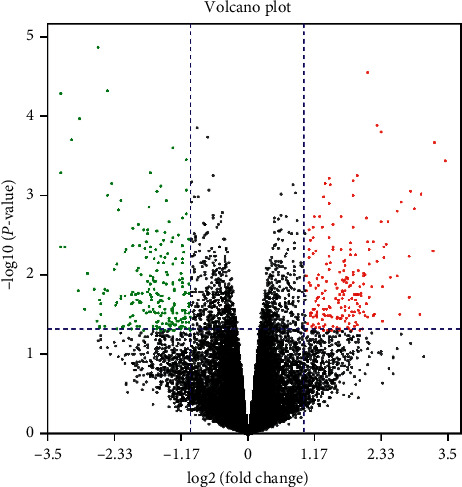
Identification of DEGs between PHN and normal control DRG samples.

DEGs were shown in Volcano diagrams (Figure 1). A total of 116 genes were up-regulated in DRGs of PHN rats and 135 genes were down-regulated.

### 3.2. KEGG and GO Analysis of DEGs

**Figure 2:**
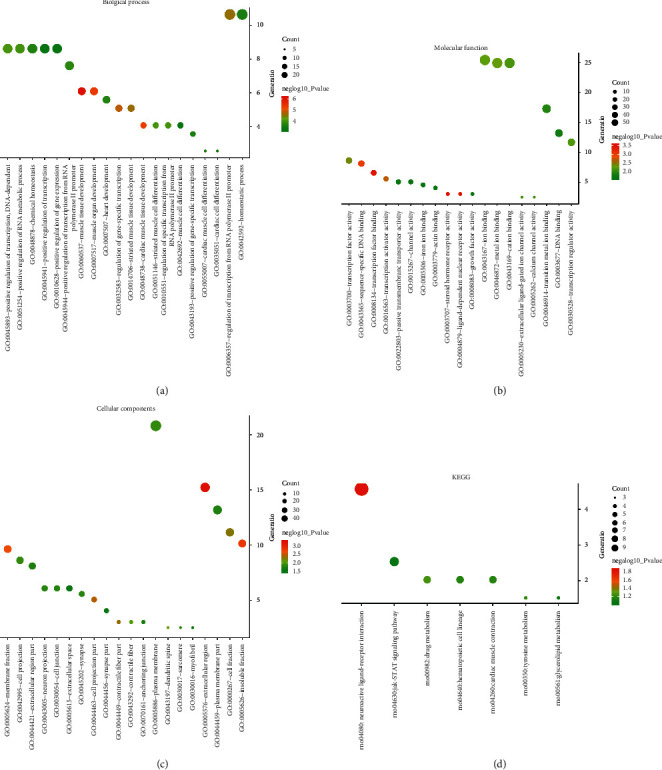
The enrichment analysis of DEGs by DAVID. The bubble charts showed (a) BP, (b) CC, (c) MF, and (d) KEGG, respectively.

**Figure 3:**
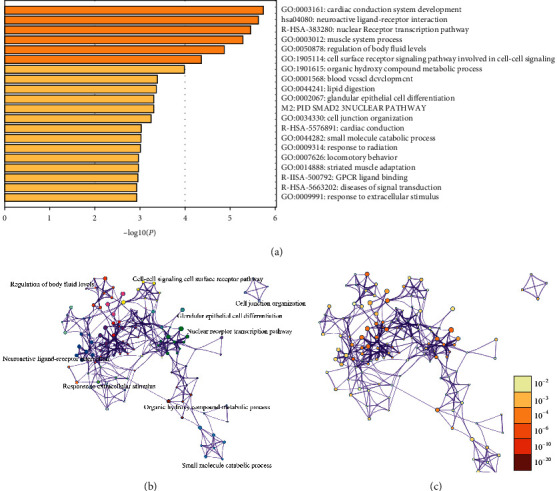
The enrichment analysis of DEGs by Metascape. (a) Bar graph of enriched terms across DEGs, colored by *P* values. (b) Network of enriched terms, colored by cluster. (c) Network of enriched terms, colored by significant *P* value.

The results of GO and KEGG analysis revealed that variations were predominantly enriched in muscle tissue development, cardiac muscle tissue development, regulation of gene-specific transcription, neuroactive ligand-receptor interaction, Jak-STAT signaling pathway, cell surface receptor signaling pathway involved in cell-cell signaling, calcium channel activity, and so on. Results from DAVID analysis were shown in Figures 2(a)–2(d). Pathway and process enrichment analysis by Metascape were shown in Figures 3(a)–3(c).

### 3.3. PPI Network

**Figure 4:**
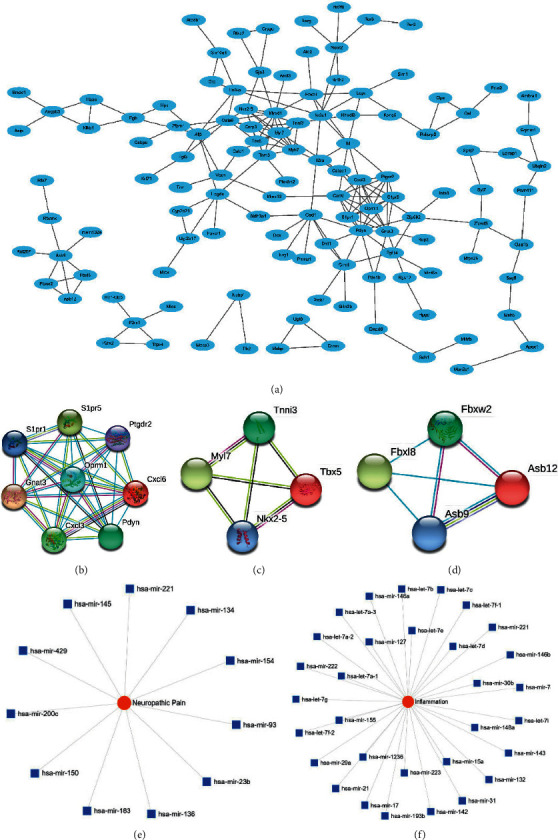
Protein-protein interaction (PPI) network and MCODE genes. (a) Protein-protein interaction network of DEGs. (b–d) Significant modules. (e) Neuropathic pain-related miRNAs. (f) Inflammation-related miRNAs.

Construction of a PPI network revealed 120 nodes and 191 edges (Figure 4(a)). Significant modules were identified (Figure 4(b)–4(d)).

### 3.4. Neuropathic Pain and Inflammation-Related miRNAs and Genes Prediction

11 miRNAs were found related with neuropathic pain (Figure 4(e)). 31 miRNAs were found related with inflammation (Figure 4(f)). In addition, 2843 genes were predicted by the neuropathic pain-related miRNAs (Supplementary Figure S1 A). 5199 genes were predicted by the inflammation-related miRNAs (Supplementary Figure S1 B). There were some intersection genes between DEGs of GSE64345 and the predicted genes by the miRNA including MCODE gene S1PR1, OPRM1, PTGDR2, and FBXW2 (Supplementary Figure S1 C, D). Furthermore, a total of 10545 genes were found associated with PHN in CTD. There were some intersection genes between DEGs of GSE64345 and the PHN-related genes, including MCODE-identified genes S1PR1, OPRM1, PDYN, CXCL3, S1PR5, TBX5, TNNI3, and MYL7 (Supplementary Figure S1 E, F).

### 3.5. Hub Gene Selection

The intersecting MCODE-identified genes were selected as hub genes: S1PR1, OPRM1, PDYN, CXCL3, S1PR5, TBX5, TNNI3, MYL7, PTGDR2, and FBXW2. The list of fold change of the expression of hub genes in PHN models compared with control was shown in Table 1.

### 3.6. CTD Analysis

**Figure 5:**
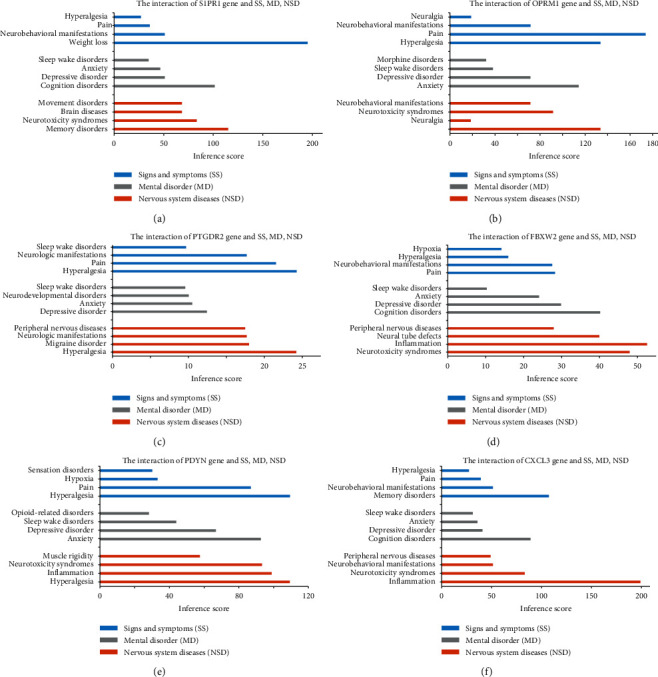
The hub gene analysis in CTD. (a) S1PR1, (b) OPRM1, (c) PTGDR2, (d) FBXW2, (e) PDYN, and (f) CXCL3.

The hub genes were analyzed in CTD, and the genes were significantly related with some signs and symptoms, mental disorder, and nervous system diseases including hyperalgesia and depression. The results suggesting that these genes may be involved in the occurrence and development of PHN. The interaction of S1PR1, OPRM1, PTGDR2, FBXW2, PDYN, and CXCL3 and inference score were shown in Figures 5(a)–5(f). In addition, we analyzed the intersecting gene between DEGs of GSE64345, PHN-related genes, and genes inferred with common drugs for treating PHN including pregabalin, gabapentin, amitriptyline, duloxetine, venlafaxine, and tramadol (Supplementary Figure S2 A-F).

### 3.7. Brain Localization and Expression

**Figure 6:**
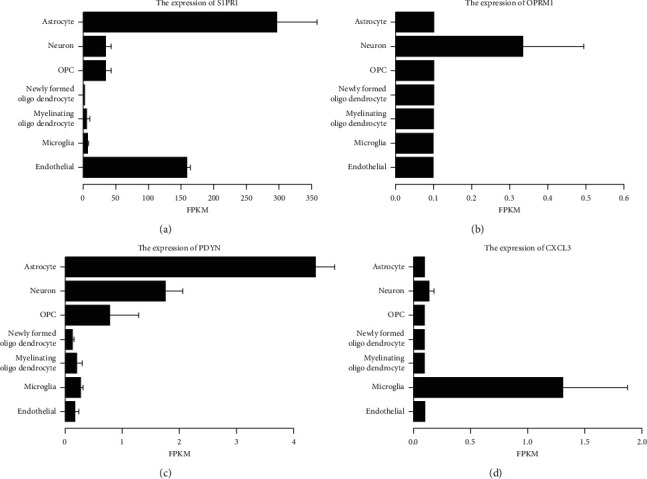
The hub gene expression and localization in brain cells. (a) S1PR1, (b) OPRM1, (c) PDYN, and (d) CXCL3.

S1PR1 and PDYN were mainly located in the astrocyte. OPRM1 was mainly located in the neuron. CXCL3 was mainly located in the microglia (Figures 6(a)–6(d)).

### 3.8. miRNA-mRNA Network and lncRNA Prediction

**Figure 7:**
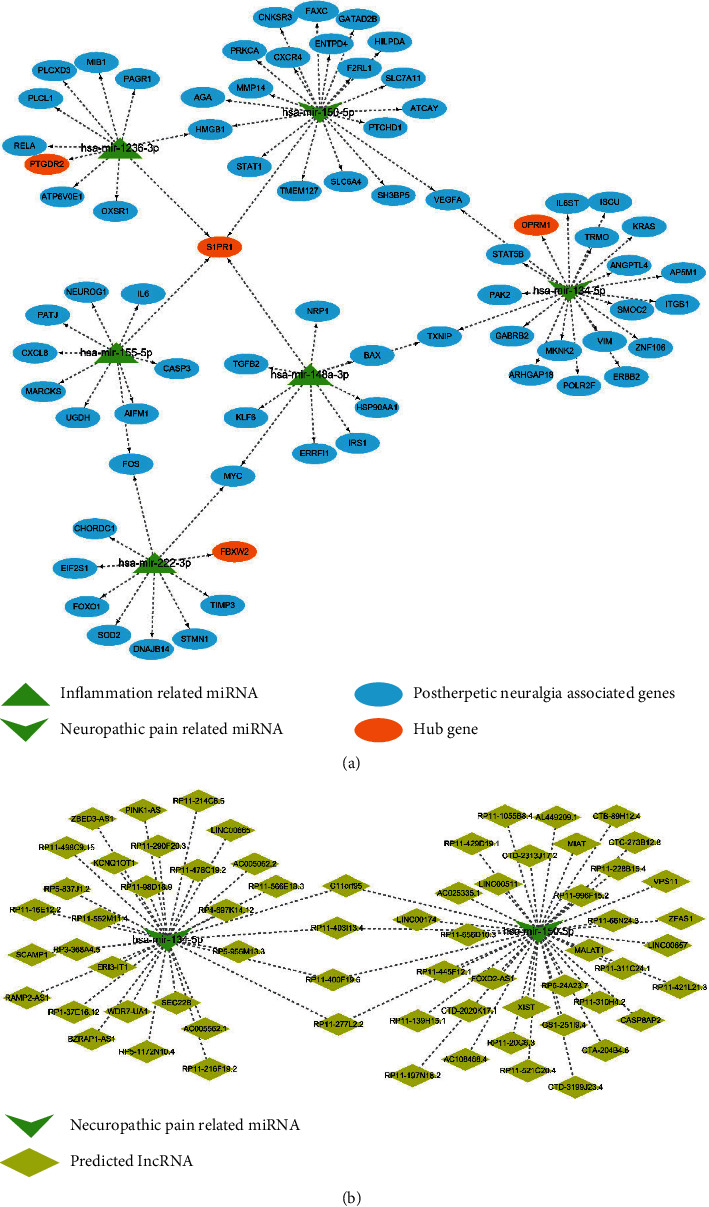
The miRNA-mRNA network and lncRNA-miRNA network. (a) miRNA-mRNA network. (b) lncRNA-miRNA network predicted by neuropathic pain-related miRNA hsa-mir-150-5p and hsa-mir-134-5p.

The neuropathic pain-related miRNA hsa-mir-150-5p, hsa-mir-134-5p, and inflammation-related miRNA hsa-mir-148a-3p, hsa-mir-155-5p, hsa-mir-1236-3p, and hsa-mir-222-3p were included in the network (Figure 7(a)). In addition, the neuropathic pain-related miRNAs hsa-mir-150-5p and hsa-mir-134-5p-predicted lncRNAs were identified, and the lncRNA-miRNA networks were constructed (Figure 7(b)).

## 4. Discussion

Elderly patients are prone to postherpetic neuralgia (PHN) following VZV-induced herpes zoster [25]. Thoracic, cervical, and trigeminal nerves are commonly involved in PHN [26, 27], which may cause anxiety, depression, sleep disorders, and other complications. As a result, the life quality of elderly patients was seriously reduced [28]. In addition, patients may be haunted by PHN for a long time. A survey of 385 patients with PHN found that the average duration was 3.3 years [29]. Therefore, PHN not only causes pain in patients but also greatly increases the burden of medical care [30]. However, the pathogenesis of PHN has not been fully elucidated, resulting in which the current treatments are far from satisfactory [10]. Therefore, it is of important clinical and market value to explore the molecular mechanism of PHN and identify better therapeutic targets.

The DRG may play an important role in the onset, development, and treatment of PHN [9, 31]. In this study, the whole genome sequencing data of DRG of PHN rats was used to identify several hub genes (S1PR1, OPRM1, PTGDR2, FBXW2, PDYN, CXCL3, S1PR5, TBX5, TNNI3, and MYL7). In addition, miRNA-mRNA networks were constructed. The roles of S1PR1, OPRM1, PTGDR2, FBXW2, PDYN, and CXCL3 in the pathogenesis of PHN are discussed.

S1PR1 (sphingosine-1-phosphate receptor 1) is mainly involved in the regulation of G protein-coupled receptor binding, sphingosine-1-phosphate receptor signaling pathway, cytokine-mediated signaling pathway, transmission of nerve impulse, neuron differentiation, actin cytoskeleton reorganization, and positive regulation of cytosolic calcium ion concentration involved in phospholipase C-activating G protein-coupled signaling pathway [32]. Song found that there was a significant correlation between S1PR1 and chemotherapeutic drug resistance in gastric cancer, which provided evidence for the study of the mechanism and treatment of chemotherapeutic drug resistance [33]. Liu demonstrated that S1PR1 can regulate the proliferation and apoptosis of esophageal cancer cells [34]. In addition, Xie found that pain sensitivity was improved in S1PR1 knockout rats. Further analysis showed that S1PR1 receptors in DRG were involved in pain sensitivity by regulating inflammation [35]. Furthermore, Grenald found that inhibition of S1PR1 could reduce cancer-induced bone pain (CIBP) and promote the expression of IL-10 in lumbar spinal cord, suggesting that S1PR1 is involved in the regulation of CIBP and neuroinflammation [36]. Similarly, Huang believed that S1PR1 might play an important role in the pathophysiology of pain and neuroinflammation [37]. Consistent with the above study, we found that S1pr1 was differentially expressed in the DRG of PHN rats. At the same time, we found that S1PR1 was the target gene predicted by both neuropathic pain-related miRNA hsa-mir-150-5p and inflammation-related miRNA hsa-mir-1236-3p, hsa-mir-155-5p, and hsa-mir-148a-3p. CTD analysis determined that S1PR1, located in astrocytes, was associated with PHN. We speculate that S1PR1 is involved in the occurrence and development of PHN by regulating inflammation and pain transmission pathway in DRG. This suggests that S1PR1 may be a specific therapeutic target for PHN, and the related molecular mechanism is worthy of further exploration.

OPRM1 (opioid receptor mu 1) is mainly involved in regulating voltage-gated calcium channel activity, morphine receptor activity, neuropeptide binding, cytokine-mediated signaling pathway, sensory perception of pain, and excitatory postsynaptic potential [32]. OPRM1 plays an important role in pain modulation and analgesia. Through the analysis of patients with primary dysmenorrhea, Wei proved that there was an association between OPRM1 gene polymorphism and descending pain modulation system (DPMS), suggesting that OPRM1 may be involved in central sensitization [38]. Chidambaran found through a retrospective analysis that DNA methylation on the OPRM1 promoter can predict pain after spinal fusion and guide medication, suggesting that OPRM1 plays an important role in pain transmission [39]. Similarly, Mo found that OPRM1 participates in the abnormal expression of pain pathway regulated by methyl CpG binding domain protein, which promotes the occurrence and maintenance of neuropathic pain [40]. In addition, there is a correlation between OPRM1 gene polymorphism and the efficacy of analgesics [41]. However, there are few reports on the relationship between OPRM1 and PHN. We found that Oprm1 was abnormally expressed in the DRG of PHN rats. Furthermore, OPRM1 is the target gene predicted by neuropathic pain-related miRNA hsa-mir-134-5p. OPRM1 was associated with PHN analyzed in CTD. We speculate that OPRM1 induces PHN by regulating pain signal transduction in DRG and central sensitization, but the molecular mechanism needs further exploration.

PTGDR2 (prostaglandin D2 receptor 2) is mainly involved in regulating neuropeptide binding, immune response, and calcium-mediated signaling. Zhang found that PTGDR2 was abnormally expressed in gastric cancer, which was related to the poor prognosis of patients [42]. In addition, Kiely found that PTGDR2 was abnormally expressed in the microglia of degenerative brain atrophy and may be involved in the inflammatory changes of degenerative brain atrophy [43]. FBXW2 (F-box and WD repeat domain containing 2) plays a role in the regulation of protein polyubiquitination, cellular protein modification process, proteolysis. Yang found that FBXW2 can participate in the occurrence and development of lung cancer by regulating epidermal growth factor-AKT1, *β*-catenin [44]. There are few reports related to FBXW2 involving in pain regulation. We found that PTGDR2 and FBXW2 were differentially expressed in DRG of PHN rats. Furthermore, PTGDR2 is the target gene predicted by inflammation-related miRNA hsa-mir-1236-3p. FBXW2 is the target gene predicted by inflammation-related miRNA hsa-mir-222-3p. We hypothesize that PTGDR2 and FBXW2 are involved in the occurrence and development of PHN by regulating inflammation and pain signal transduction in DRG.

PDYN (prodynorphin) is mainly involved in regulating opioid peptide activity, neuropeptide signaling pathway, chemical synaptic transmission, and sensory perception. Rojewska found abnormal expression of PDYN in the spinal cord using the mouse model of chronic sciatic nerve compression injury (CCI), suggesting that PDYN may play an important role in the occurrence and development of neuropathic pain [45]. Similarly, Korczeniewska examined the DRG and trigeminal ganglion (TG) of trigeminal neuropathy and spinal mononeuropathy induced by chronic compression injury. Pdyn was up-regulated in TG and down-regulated in DRG 4 days after injury [46]. CXCL3 (C-X-C motif chemokine ligand 3) is involved in regulating chemokine activity, chemokine-mediated signaling pathway, inflammatory response, neutrophil chemotaxis, and immune response. Liu found that CxCl3 was highly expressed in the cervical spinal cord of pruritus animal model, suggesting that CxCl3 may mediate pruritus [47]. In addition, Piotrowska proved that CXCL3 may mediate the process of neuropathic pain and hyperalgesia [48]. Consistent with these results, we found that PDYN and CXCL3 were differentially expressed in the DRG of PHN rats. We speculate that PDYN and CXCL3 induce and maintain PHN by regulating DRG pain conduction pathway and central sensitization. The related molecular mechanisms need additional investigation.

Despite our rigorous analysis, there are several shortcomings in this study. Firstly, the sample size in the dataset is small, and a larger sample size is needed to yield more accurate results. Secondly, the expression of the hub genes in the DRG has not been verified. Thirdly, we speculated the functional pathway of hub genes involved in the pathogenesis of PHN and explored the molecular targets of gabapentin and other drugs used in the treatment of PHN. However, the specific molecular mechanisms of these hypotheses must be verified by further experiments.

## 5. Conclusions

Bioinformatics analysis is a useful tool to explore the mechanism and pathogenesis of PHN. There were numerous genes that were differentially expressed in the DRG of PHN rats and normal control groups. These hub genes may play important roles in the onset and development of PHN and serve as therapeutic targets.

## Figures and Tables

**Table 1:** The information of 10 hub gene.

Gene symbol	Title	Log FC	*P* value
Oprm1	Opioid receptor, mu 1	1.09	0.03269922
Cxcl3	Chemokine (C-X-C motif) ligand 3	1.79	0.00471944
Tnni3	Troponin I3, cardiac type	1.83	0.00244407
Tbx5	T-box 5	3.24	0.00484397
Ptgdr2	Prostaglandin D2 receptor 2	−1.32	0.00724425
Myl7	Myosin light chain 7	−1.33	0.02720756
Fbxw2	F-box and WD repeat domain containing 2	−1.8	0.0046277
Pdyn	Prodynorphin	−1.48	0.00687388
S1pr1	Sphingosine-1-phosphate receptor 1	−2.07	0.04005879
S1pr5	Sphingosine-1-phosphate receptor 5	−2.13	0.00474774

FC: fold change.

## Data Availability

The data of this research were downloaded from the public GEO database. The additional data used to support the findings of the study are available from the corresponding author upon request.

## Abbreviations

S1PR1: Sphingosine-1-phosphate receptor 1
OPRM1: Opioid receptor, mu 1
PDYN: Prodynorphin
CXCL3: Chemokine (C-X-C motif) ligand 3
S1PR5: Sphingosine-1-phosphate receptor 5
TBX5: T-box 5tbox5
TNNI3: Troponin I3, cardiac type
MYL7: Myosin light chain 7
PTGDR2: Prostaglandin D2 receptor 2
FBXW2: F-box and WD repeat domain containing 2.

## Supplementary Materials

Supplementary Materials Supplementary Figure S1. (A-B) Genes predicted by the neuropathic pain and inflammation-related miRNAs; (C-D) Intersection genes between DEGs and the predicted genes; (E-F) Intersection genes between DEGs, MCODE genes, and the PHN-associated genes. Supplementary Figure S2:Analysis of PHN-associated genes, drug-related genes, and DEGs. (A) Pregabalin, (B) Gabapentin, (C) Amitriptyline, (D) Duloxetine, (E) Venlafaxine, (F) Tramadol.
