# Supplementary material for: Bioinformatics Analysis of Genes and Mechanisms in Postherpetic Neuralgia
Source: Pain Res Manag. 2020 Sep 24;2020:1380504. doi: 10.1155/2020/1380504 (PMC7532419; doi:10.1155/2020/1380504)

Supplementary Figure S1: (A-B) Genes predicted by the neuropathic pain and inflammation related miRNAs; (C-D) Intersection genes between DEGs and the predicted genes; (E-F) Intersection genes between DEGs, MCODE genes and the PHN associated genes.


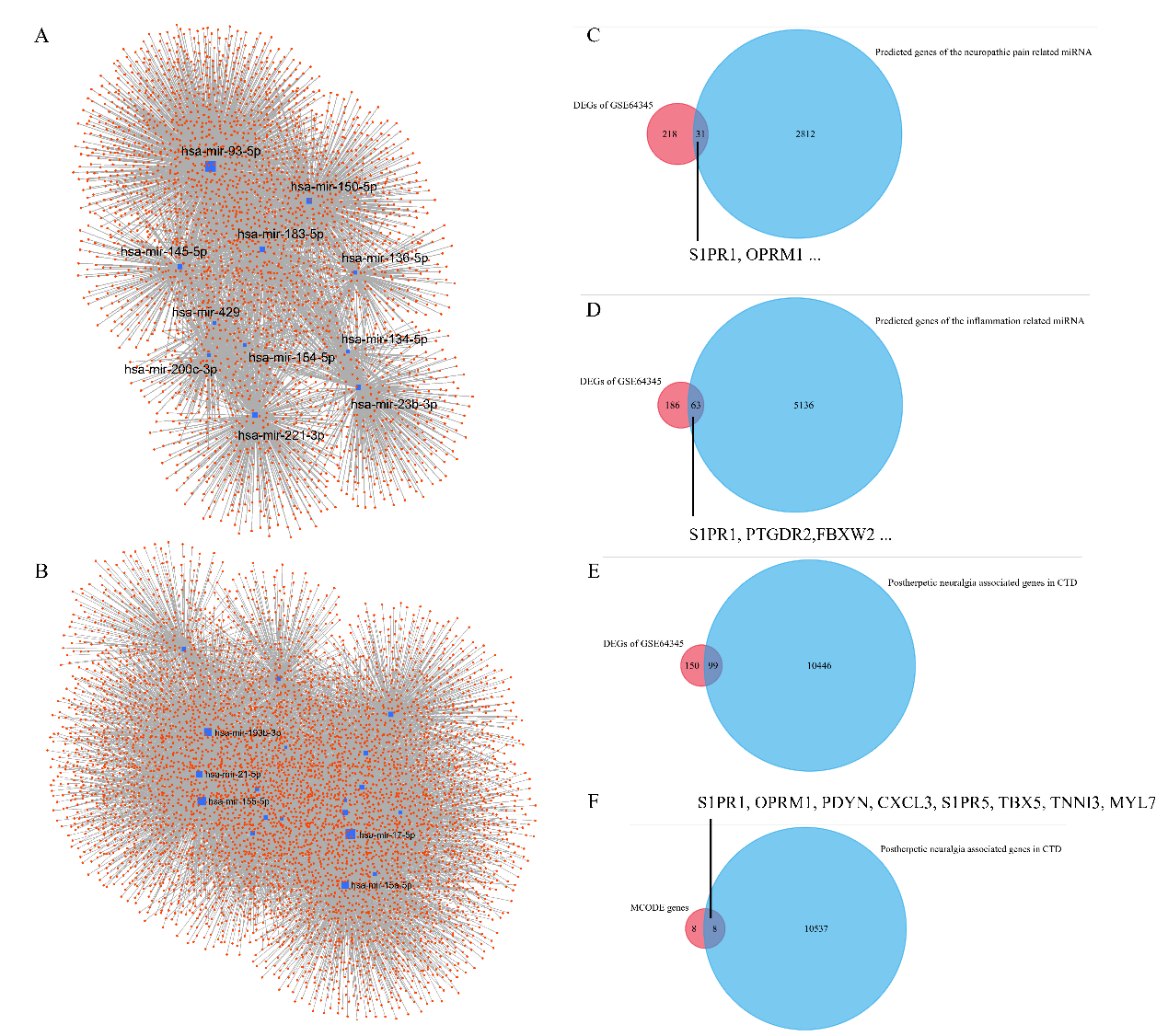


Supplementary Figure S2: Analysis of PHN associated genes, drug related genes and DEGs. (A) Pregabalin, (B) Gabapentin, (C) Amitriptyline, (D) Duloxetine, (E) Venlafaxine, (F) Tramadol.


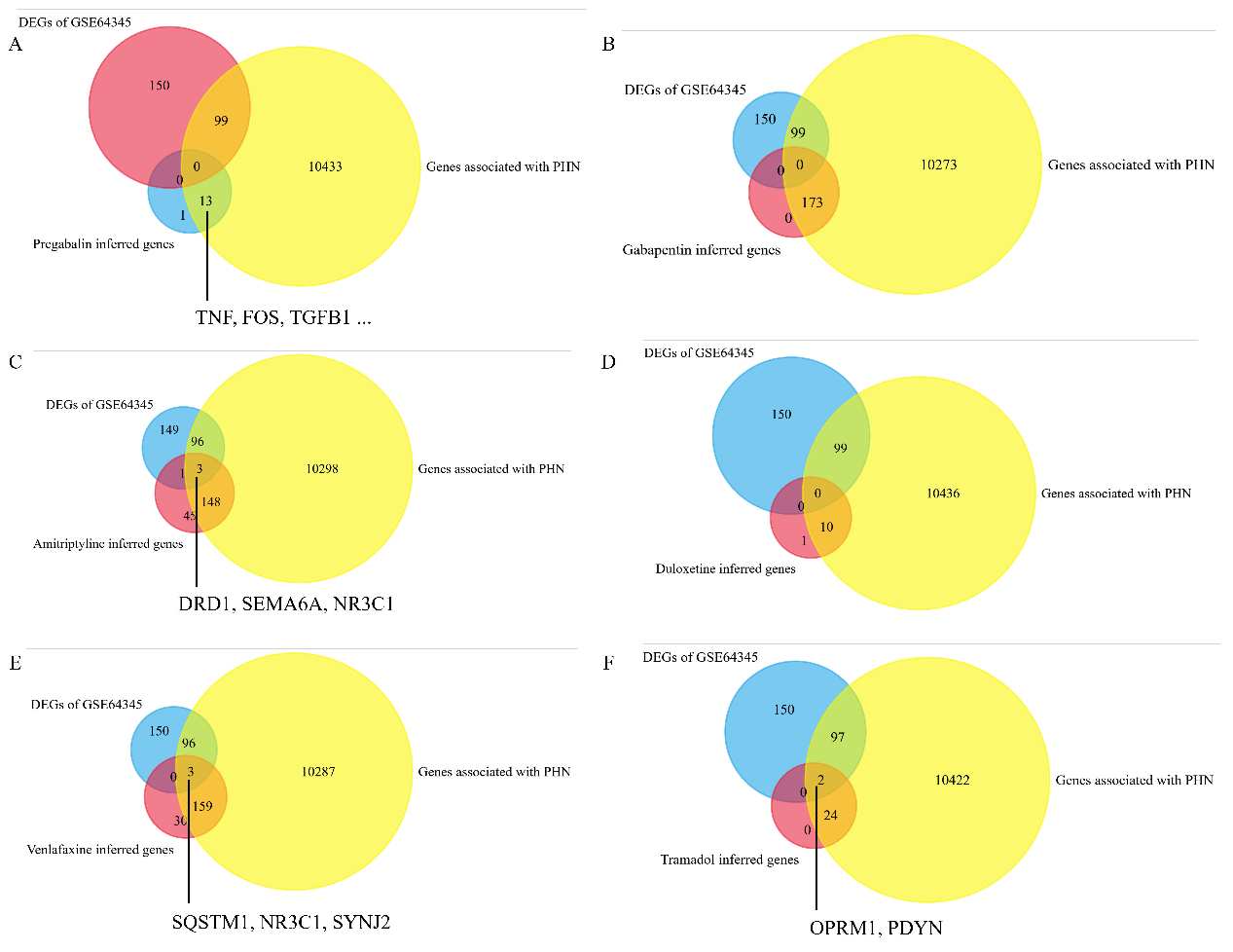

Supplement: Supplementary Materials — Supplementary Figure S1. (A-B) Genes predicted by the neuropathic pain and inflammation-related miRNAs; (C-D) Intersection genes between DEGs and the predicted genes; (E-F) Intersection genes between DEGs, MCODE genes, and the PHN-associated genes. Supplementary Figure S2:Analysis of PHN-associated genes, drug-related genes, and DEGs. (A) Pregabalin, (B) Gabapentin, (C) Amitriptyline, (D) Duloxetine, (E) Venlafaxine, (F) Tramadol. [file 1380504.f1.docx]
